# Gunshot Fractures of the Long Bones of the Extremities

**Published:** 1918-12

**Authors:** 


### Gunshot Fractures of the Long Bones of the Extremities.

By First Lieut. HAROLD FROST. *The Military Surgeon*, June, 1918.

*Postural Treatment of Fractures.* This must be carried on together with the treatment of infection, whenever it is possible. The desired aim is to obtain a firmly united bone shaft, with a fair degree of alignment, and as little shortening as possible. Two methods of splinting are used : the external, by means of apparatus; the internal, by means of plates, non-absorbable sutures, and bone grafts.

*External Apparatus.* As yet no method of splinting has been devised so efficient as to be generally adopted. A certain number of qualities are required altogether for the good reducing of the fractures, the possibility of giving necessary treatment to the wounds and soft parts, and the comfort of the patient.

Fractures can be divided into non-infected and infected fractures.

*Non-infected Fractures.* They are always simple. Absolute immobility is essential for two weeks to prevent infection from developing along the track of the missile.

*Fore-Arm.* All cases were limited to fractures of one bone only: the radius or ulna. A simple fixation splint is sufficient. If the wound involves the dorsum of the fore-arm alone, the author uses *Osgood-Penhallow* tin splint. If the wound involves the anterior surface a fenestrated plaster may be used. Such fractures should remain in apparatus from 4 to 5 weeks.

*Humerus.* Light plaster proves very useful, including shoulder, arm, fore-arm to the wrist and circular around the body. The arm is abducted at 20 degrees, the fore-arm flexed at right angles. If an undue shortening is present the *Osgood-Penhallow* splint may be used. Immobilization should last from 4 to 6 weeks.

*Femur.* There is generally a considerable shortening. This is reduced by the *Jones* abduction frame, if the fracture is in the upper third of the femur; by traction with suspension if the fracture is lower down.

*Leg.* The best and simplest apparatus is the plaster from groin to toe, with windows cut out over the wounds.

*Infected fractures. Fore-Arm. Osgood-Penhallow* splint is useful with non-perforating wounds of the dorsal aspect. In the case of extensive lateral wounds, the author prefers the use of a wire suspension splint, with transverse supporting rubber bands. The supporting bands are attached by clips, thus they can be removed for dressings. In the case of an antero-posterior perforation, three splints are used.

Shortening occurs generally when both bones are fractured. In such cases, the wire suspension with a 3 or 4 pound traction on its extremity proves very convenient.

*Arm.* The author treats such fractures in bed for the first weeks; and finds the wire suspension splint most useful. The traction is applied by straps to the fore-arm, either of adhesive, or of gauze or flannel attached to the skin by *Hauser*'s glue.

In cases presenting some inflammation the *Osgood-Penhallow* splint is adaptable. It is especially useful for an ambulatory splint.

*Femur.* Again for fractures of the upper third, the author uses the *Jones* abduction frame, and a wire suspension splint for fractures lower down. Traction varying from 15 to 25 pounds is applied to the leg and uninjured parts of the thigh by adhesive straps.

*Leg.* Fractures with undue shortening may be treated in the wire suspension splint. Traction is provided as formerly. If no shortening interferes, the author has found the *Cabot* wire splint most convenient.

*Internal Splints.* The plating of infected bone fracture has been very much objected to. By the incisions necessary for exposing the bone shaft, fresh tissues are laid open to infection; through the screwholes new foci of osteomyelitis develop, and because of this additional necrosis convalescence is prolonged. The plates loosen often before sufficient union between the bone fragments has developed and in all cases act as a foreign body prolonging suppuration.

The adepts of this method of course deny these disadvantages and assert that the plating is followed by good effects : the pain caused in the transport is entirely avoided, the functional results are better, the dressings are easily done, massage and joint motions can take place sooner.

Non-absorbable sutures, either metal or animal, can be criticized in the same way.

As for bone grafts, they may be of use in cases of large gaps in the bone shaft. With a mild infection, the graft may be tolerated, and even osteogenesis may be stimulated. In severe infections, the graft will generally loosen, and be extruded.

*Complications. Muscles and Tendon Adhesions.* They generally cannot be avoided in very severe cases; otherwise massage, electrical stimulation, and movements of tendons and joints prove effective.

But this must not be undertaken until infection is quite controlled, and bone union has begun.

If adhesions intrude in spite of treatment, the use of *Turner* apparatus often restores the function by gradual stretching of the adhesion.

*Nerve Injuries.* Immediate lesions consist of complete and incomplete divisions, contusions, and concussions. Remote lesions consist of compression. These should be operated upon as soon as possible; the injured nerve released and placed in a new sheath.

*Pseudo-arthrosis.* The only method of treatment is generally the insertion of an autogenous bone graft.

*Secondary Hemorrhage.* It happens rarely, and arises from arterial source in infected wounds. The treatment consists of immediate operation : securing and tying the bleeding vessel near the point of rupture.

